# The RGD Domain of Human Osteopontin Promotes Tumor Growth and Metastasis through Activation of Survival Pathways

**DOI:** 10.1371/journal.pone.0009633

**Published:** 2010-03-10

**Authors:** Donald Courter, Hongbin Cao, Shirley Kwok, Christina Kong, Alice Banh, Peiwen Kuo, Donna M. Bouley, Carmen Vice, Odd Terje Brustugun, Nicholas C. Denko, Albert C. Koong, Amato Giaccia, Quynh-Thu Le

**Affiliations:** 1 Department of Radiation Oncology, Stanford University, Stanford, California, United States of America; 2 Department of Pathology, Stanford University, Stanford, California, United States of America; 3 Department of Comparative Medicine, Stanford University, Stanford, California, United States of America; 4 Department of Oncology, Oslo University Hospital – Norwegian Radium Hospital, Oslo, Norway; Roswell Park Cancer Institute, United States of America

## Abstract

**Background:**

Human osteopontin (OPN), a known tumor associated protein, exists in different isoforms, whose function is unclear. It also possesses a RGD domain, which has been implicated in diverse function. Here, we use genetic approaches to systematically investigate the function of the RGD domain in different OPN isoforms on tumor progression and metastasis for 2 different solid tumor models.

**Methodology/Principal Findings:**

Using isoform-specific qRT-PCR, we found that OPN-A and B were the main isoforms overexpressed in evaluated human tumors, which included 4 soft tissue sarcomas, 24 lung and 30 head and neck carcinomas. Overexpression of either OPN-A or B in two different cell types promoted local tumor growth and lung metastasis in SCID mouse xenografts. However, expression of either isoform with the RGD domain either mutated or deleted decreased tumor growth and metastasis, and resulted in increased apoptosis by TUNEL staining. *In vitro*, whereas mutation of the RGD domain did not affect cell-cell adhesion, soft agar growth or cell migration, it increased apoptosis under hypoxia and serum starvation. This effect could be mitigated when the RGD mutant cells were treated with condition media containing WT OPN. Mechanistically, the RGD region of OPN inhibited apoptosis by inducing NF-κB activation and FAK phosphorylation. Inhibition of NF-κB (by siRNA to the p65 subunit) or FAK activation (by a inhibitor) significantly increased apoptosis under hypoxia in WT OPN cells, but not in RGD mutant cells.

**Conclusion/Significance:**

Unlike prior reports, our data suggest that the RGD domain of both OPN-A and B promote tumor growth and metastasis mainly by protecting cells against apoptosis under stressed conditions and not via migration or invasion. Future inhibitors directed against OPN should target multiple isoforms and should inhibit cell survival mechanisms that involve the RGD domain, FAK phosphorylation and NF-κB activation.

## Introduction

Osteopontin (OPN) is a phosphoglycoprotein that has important physiological functions in bone remodeling [1], immune response and inflammation [2], [3]. It is also a tumor-associated protein, which mediates tumor transformation and malignant progression [4], [5]. Elevated OPN levels have been shown to correlate with increased tumor progression and poor survival in many solid tumors [6]. OPN has been proposed to promote tumor progression through several mechanisms, including increased cell survival, migration, invasion, neovascularization, and modulation of immune function [7].

When OPN was first identified and sequenced from a rat osteosarcoma phage library, it was found to contain the RGD sequence that is known to be present on fibronectin and binds to integrins [8]. Therefore, it was hypothesized that the RGD domain of OPN functionally mediates cell adhesion, migration and invasion through integrin engagement. Further studies confirmed these proposed functions of the OPN RGD domain in osteoblasts, endothelial cells and certain cancer cell lines [5]. However, most of these studies employed recombinant OPN (which lacks post-translational modifications), competing synthetic RGD peptides or their mutated versions, and αvβ3 integrin blocking antibodies to investigate the role of the RGD domain [9], [10], [11], [12], [13]. Some studies used purified OPN derived from non-cancer sources such as breast milk, bones, osteoblasts or smooth muscle cells [14], [15], [16], [17]. However, recent data have shown that the pattern of OPN post-translational modification, specifically phosphorylation, is cell-type dependent and may affect the degree of cell adhesion to this molecule [4], [18], [19], [20]. For example, OPN from *ras*-transformed mouse fibroblasts has less phosphorylation than OPN from differentiated mouse osteoblasts and can bind more readily to MDA435, a breast cancer cell line [19]. Similarly, tumor derived OPN has been shown to exist in a secreted form, not bound to the extracellular matrix, and unable to support cell adhesion when added to the condition media [21]. Therefore, the question of whether the RGD domain of tumor-derived OPN indeed mediates cell adhesion and migration has not been directly addressed. Only one study has attempted to answer this question by overexpressing full-length OPN-B splice form and the same construct with the RGD domain deleted in one breast cancer cell line [22]. This study revealed that loss of the RGD region partially decreased lymphatic metastasis in a breast cancer model, however, deletion of the entire domain may result in unpredictable structural effects that can interfere with function.

Another layer of added complexity is that human OPN gene is subject to alternative splicing, which yields three distinct isoforms: OPN-A (full-length transcript), OPN-B (lacks exon 5 or 14 amino acids) and OPN-C (lacks exon 4 or 28 amino acids) [23]. The relative expression of the 3 isoforms in human tumors and their function is not well characterized. While one study suggested that OPN-C was selectively expressed in invasive breast cancer and more likely to support anchorage independent tumor growth than OPN-A [24], another study indicated that OPN-C was less likely to promote cell migration and invasion than the other 2 OPN isoforms in mesothelioma cells [25]. These differences in OPN isoform expression have not consistently been investigated.

To address the function of the RGD domain in tumor-derived OPN for different splice forms and in the different human tumor cell lines, we overexpressed (1) wild type OPN (WT), (2) OPN with mutations in the RGD domain (RAA), or (3) OPN with the RGD deleted (NoRGD) for the two different splice forms: OPN-A and OPN-B. We have previous analyzed several primary human cancers, including soft tissue sarcomas (STS), non-small cell lung cancers (NSCLC) and head and neck squamous cell carcinomas (HNSCC) and found that OPN-A and B but not C had elevated expression in these tumors compared to matched normal tissues. Overexpression of either the WT OPN-A or OPN-B isoform resulted in enhanced tumor growth and metastatic lung colony formation in two cell types. This effect appeared to be mediated through the RGD segment since either mutation or deletion of this domain abolished OPN-enhancement of tumor growth or metastasis. Comparison of RGD-mutated and RGD-WT tumors indicated that OPN-promotion of tumor growth was due to less apoptosis in WT cells. *In vitro* studies showed that RGD mutation did not affect cell adhesion, migration or invasion in these cell lines. However, it increased cell apoptosis under hypoxia and serum starvation when compared to WT OPN expressing cells, and this effect was partly restored with condition media enriched in WT OPN. OPN anti-apoptotic effect was signaled mainly through the activation of FAK and NF-κB on further investigation. Taken together, these results suggest that the RGD domain of tumor-derive OPN promotes tumor growth and metastasis mainly through cell survival mechanisms, involving FAK and NF-κB in our model.

## Materials and Methods

### Cell Lines & Hypoxia Treatment

MiaPaCa-2 (human pancreatic cancer cell line), HT1080 (human fibrosarcoma cell line), FaDu (human HNSCC cell line), NCI-460 (human NSCLC cell line) and MDA231 (human breast carcinoma cell line) were obtained from the American Type Culture Collection (ATCC). SCC22B (human HNSCC cell line) was obtained from the University of Michigan (Courtesy Dr. Carey). Cell lines were maintained in DMEM supplemented with 10% fetal bovine serum. For hypoxia treatments, the cells were maintained in an anoxia chamber (Sheldon Manufacturing, Cornelius, OR) with an estimated pO_2_<0.02% for specified durations.

### Antibodies

The following antibodies were used: mouse monoclonal antibodies against phospho-tyrosine 397 FAK, total FAK and human Bcl-2 (BD Biosciences, San Jose, CA), mouse monoclonal anti-XIAP antibody (Abcam, Cambridge, MA), mouse monoclonal anti-β-actin antibody (Sigma-Aldrich, St Louis, MO), and rabbit polyclonal antibodies against AKT and phospho-serine 473 AKT (Cell Signaling, Danvers, MA).

### Construction of OPN Plasmids and Transfection

Human OPN constructs were cloned into pcDNA 3.1 vector (Invitrogen, Carlsbad, CA). OPN-A was PCR amplified and OPN-A-RAA was generated by site-directed mutagenesis. OPN-B and OPN-B-NoRGD (RGD deleted) were gifts from Dr. Alison Allan (Univ. of Western Ontario) [22]. HT1080 and MiaPaCa-2 cells were transfected using Lipofectamine 2000 (Invitrogen).

### Immunoblotting

Immunoblotting was performed as previously described [26]. Briefly, cells were lysed using 9 M urea and 75 mM Tris HCl (pH 7.5) lysis buffer supplemented with 150 mM β-mercaptoethanol. Equivalent amounts of protein were denatured, electrophoresed, and transferred to membranes. After incubation/washing with the appropriate primary and secondary antibodies, proteins were detected by Amersham ECL (GE Healthcare) and autoradiography film.

### *In Vitro* Cell Proliferation, Adhesion, Soft Agar Growth, Migration, Scratch and NF-κB Luciferase Reporter Assays

All assays below were performed in serum free media whenever appropriate to ensure that no exogenous OPN was added. *In vitro* cell proliferation was assessed by cell counts with a hemacytometer. To assess *in vitro* cell adhesion, FaDu HNSCC cells expressing different OPN constructs were plated in 24-well plates and incubated overnight. Luciferase expressing FaDu cells were then added to each well and allowed to adhere for 30 or 60 minutes. After non-adherent cells were washed away, residual luciferase activity was measured. Cell-cell adhesion, measured by residual luciferase signal, was quantified as a percentage of the total luciferase activity from input luciferase expressing FaDu cells [27]. For the soft agar colony formation assay, MiaPaCa-2 cells (5×10^3^), overexpressing different OPN constructs, were plated on soft agar and grown for 10-14 days followed by crystal violet staining. The number of colonies/plate and colony size, expressed as average area, were quantified using RT-Image software [28]. The scratch assay was performed with MiaPaCa-2 cells, overexpressing various constructs. Upon growth to confluency, cells were pipet scratched, cultured in serum-free media and maintained in hypoxia for 24 hours [29]. NF-κB luciferase assays were carried out as previously described [26].

### Reverse Transcription Polymerase Chain Reaction Analysis (RT-PCR)

RNA purification and RT-PCR was performed as previously described [26]. The following oligomers were used for RT-PCR: forward 5′- TTGCTTTTGCCTCCTAGGCA-3′ and reverse 5′-GTGAAAACTTCGGTTGCTGG-3′.

### OPN ELISA

OPN ELISAs were performed as previously described [30].

### Tumor Growth and Metastasis

All animal studies were approved by the Stanford University Administrative Panel on Laboratory Animal Care (APLAC). HT1080 (2.5×10^6^ cells/injection) or MiaPaCa-2 (2×10^6^ cells/injection) cells expressing different OPN constructs were implanted into the flanks of SCID mice. Tumors were measured every 2–3 days. Tumor volume was calculated by the formula (π x length x width x height)/6. For metastasis formation, HT1080 (1×10^6^ cells/injection) or MiaPaCa-2 (5×10^5^ cells/injection) cells expressing different OPN constructs were injected into the tail vein of SCID mice. After 6 weeks, lung tissue was inflated, fixed, embedded, sectioned, and stained with hematoxylin and eosin (H&E). Tumor colonies per lung section were quantified by microscopy.

### Histologic Studies

The In Situ Cell Death Detection Kit (Roche, Pleasanton, CA) was used for apoptosis evaluation on paraffin embedded HT1080 and frozen MiaPaCa-2 tumor sections according to the manufacturer's protocol. Sections were also stained with anti CD31 (Santa Cruz Biotechnology, Santa Cruz, CA), Ki67 antibody (Dako North American, Carpinteria, CA), and CD11b antibody (BD PharMingen, San Diego, CA). The human HNSCC tissue microarray was generated and stained for OPN and IKK-β as previously described [31].

### In Vitro Apoptosis Evaluation

MiaPaCa-2 and HT1080 cells overexpressing different OPN constructs were plated in 6 cm tissue culture dishes and allowed to adhere for 24 hrs. The cells were washed, permeabilized with 0.1% Triton X-100 and fixed in a 4% paraformaldehyde solution containing 10 ug/mL DAPI (Sigma-Aldrich). Apoptotic nuclei with condensed chromatin or nuclear fragmentation were assessed qualitatively using fluorescent microscopy. TUNEL(+) cells were detected using the In Situ Cell Death Detection Kit (Roche) and quantified by flow cytometry. For the *in vitro* caspase 3/7 assay, lysates were combined with a lumogenic substrate containing the DEVD sequence. Caspase activity was determined by luciferase luminescence per manufacturer instructions (Promega, Madison, WI).

### Inhibition of NF-κB

The p65 siRNA (Dharmacon, Lafayette, CO) was used to inhibit p65 per the manufacturer's protocol.

### Inhibition of FAK

PF573228 (Tocris Bioscience, Ellisville, MO) was added to cell culture media at a final concentration of 500 nM. Following a 30 minute pre-incubation, media was removed and replaced with serum-free media containing the inhibitor or vehicle. Cells were incubated in the anoxia incubator, extracts were collected, and immunoblotting was performed. Cells were also analyzed for TUNEL staining by flow cytometry.

### Statistics

Statistical analysis was performed using Statview (SAS Institute Inc, Carey, NY) software. The student T-test and the ANOVA (analysis of variance) test were use to determine the equality of the mean between different groups. Results were considered statistically significant if the p-value was <0.05.

## Results

### Expression of Different OPN Splice Variants in Human Solid Cancers

We used splice form specific primers and qRT-PCR to determine the presence of OPN-A and B in five tumor cell lines, 24 non-small cell lung cancers (NSCLC) and 30 HNSCC (Fig. S1). Supplementary Figure S1 shows representative RT-PCR for the various human solid cancer cell lines and the 4 STS. Most tumors and cell lines expressed predominantly OPN-A and B but not C. Based on these data we proceeded to assess OPN-A and B levels but not C in human NSCLC and HNSCC. Both NSCLC and HNSCC specimens (Fig. S1) expressed higher levels of OPN-A and B than normal tissues. OPN-A and B levels tracked well with total OPN gene expression on gene array analysis (data not shown). Based on these data, we focused subsequent tumor studies on OPN-A and B splice forms.

### The RGD Region Is Critical for Promoting Tumor Growth and Metastasis

**Figure 1:**
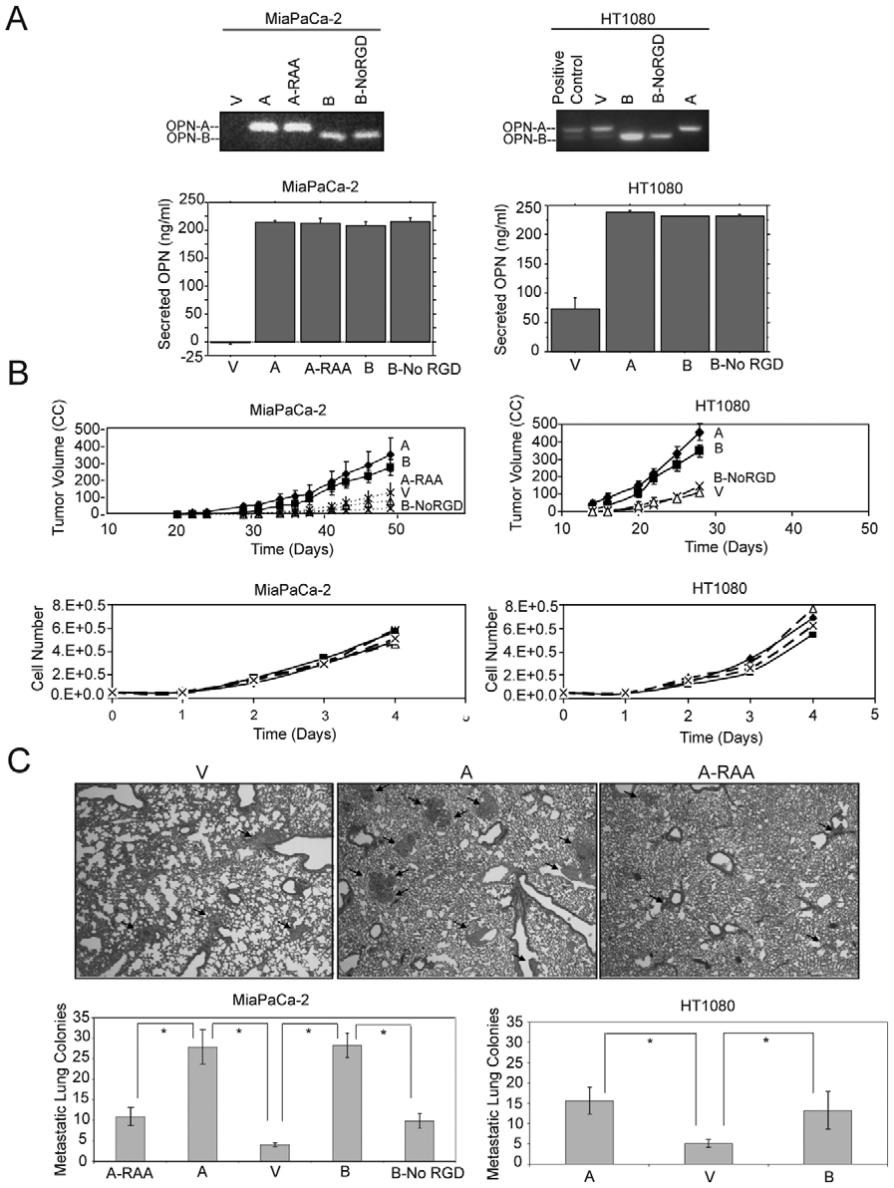
OPN promotes tumor growth and metastasis through the RGD domain. Overexpression and increased secretion of both OPN isoforms in MiaPaCa-2 (left) and HT1080 (right) cells stably transfected with OPN-A (A), OPN-B (B), OPN-A-RAA (A-RAA), OPN-B-No RGD (B-NoRGD), or pcDNA vector control (V) (Pool population) are shown (A). RT-PCR results confirmed expression of the transfected OPN constructs (A, upper panel). OPN ELISA results confirmed increased OPN secretion in transfected cells (>200 ng/mL) as compared to vector control cells (very low or undetectable levels). Secretion level was similar for all constructs (A, lower graphs). The RGD region of OPN promotes primary tumor growth in both isoforms (B). MiaPaCa-2 (left) and HT1080 (right) cells were injected into the flank of SCID mice (n = 5). OPN-A and OPN-B expressing tumors were significantly larger (B, top graphs). MiaPaCa-2 (left) and HT1080 (right) cell lines expressing OPN isoforms were plated in growth media. Similar *in vitro* growth rates were observed for all cell lines (B, bottom graphs). The RGD region of OPN mediates lung colony formation in both isoforms (C). MiaPaCa-2 Vector (left), OPN-A (middle) and OPN-A-RAA (right) cells were injected into the tail veins of SCID mice. Lung tissues were harvested 6 weeks later and stained with H&E. Metastatic lung colonies are indicated with black arrows (C, top panels). Quantification of metastatic lung colony formation in for MiaPaCa-2 (left) and HT1080 (right) cells overexpressing OPN isoforms is shown (C, bottom graphs). Stars indicate p<0.001.

Two cell lines, which have either undetectable (MiaPaCa-2) or low basal OPN expression (HT1080), were transfected with mammalian expression plasmids containing OPN-A, OPN-B, OPN-A with mutated RGD sequence (OPN-A-RAA) or OPN-B with deleted RGD sequence (OPN-B-NoRGD). A pool of cells stably overexpressing the desired OPN construct was selected. Figure 1A shows overexpression of different OPN constructs in transfected cells. Note that all OPN isoforms are secreted at similar levels.

Both MiaPaCa-2 and HT1080 cells were injected subcutaneously into SCID mice and tumor growth was monitored. Tumors transfected with WT OPN-A or B grew significantly faster than the vector control, where as those expressing OPN-A-RAA or OPN-B-NoRGD grew at a similar rate as the vector control cells (Fig. 1B). These differences in tumor growth were not due to changes in cell proliferation because the *in vitro* proliferation rates of all cell lines were similar (Fig. 1B, bottom panels).

To test the role the RGD domain in both OPN isoforms during metastatic spread, the above cells were injected into the tail veins of SCID mice. After 6 weeks, the lungs were collected and quantified for metastatic lung colonies. Control cells formed few colonies (4–5 colonies/field). However, OPN-A and OPN-B transfected cells formed more numerous and larger metastatic colonies (16 and 13 colonies/field for HT1080 & 28 colonies/field for MiaPaCa-2, respectively; Fig. 1C, p<0.001). In contrast, OPN-A-RAA and OPN-B-NoRGD expressing cells had significantly fewer colonies than the WT isoforms. These studies indicate that OPN promotes metastatic lung colony formation and loss of the RGD sequence suppresses this phenomenon. Both tumor cell types used in these assays express α_vβ3_ and β_1_ integrins and can bind to the RGD sequence (data not shown).

### OPN Decreases Apoptosis in Tumors in an RGD Dependent Manner

**Figure 2:**
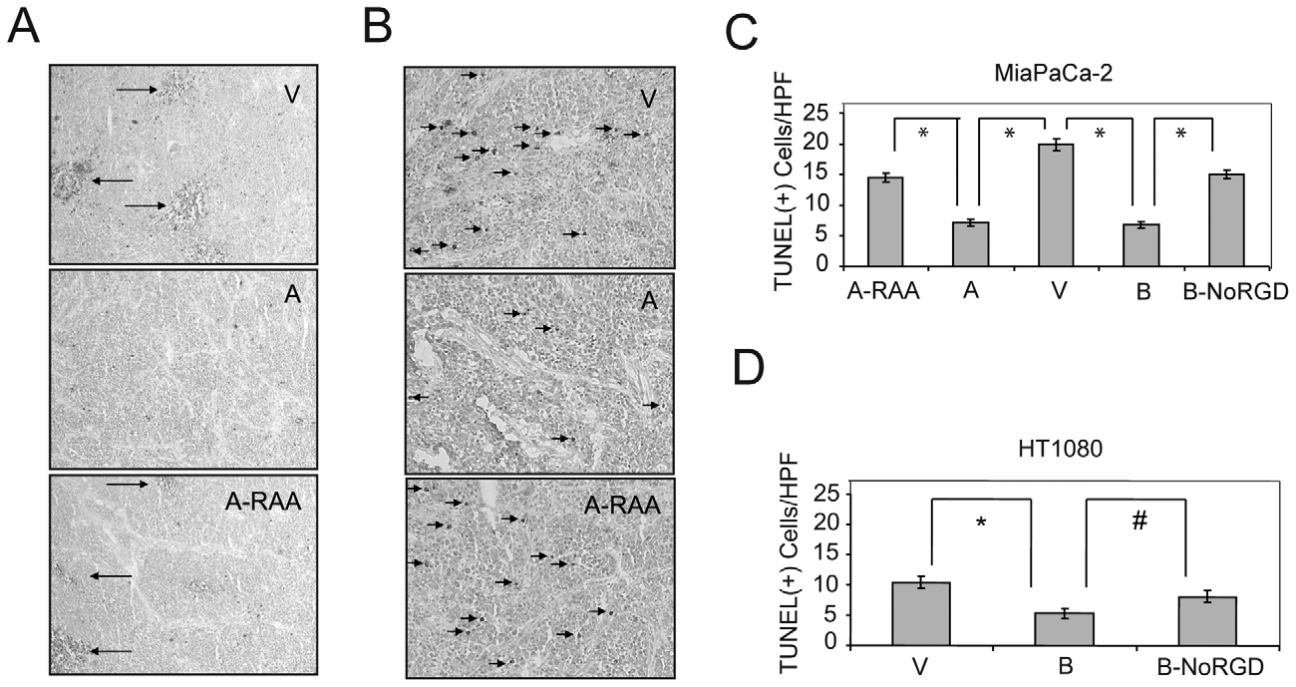
TUNEL staining in MiaPaCa-2 and HT1080 tumor tissues. MiaPaCa-2 tumors harvested 49 days after rear flank implantation in SCID mice were frozen and processed for TUNEL staining (A and B). 5× magnification is shown (A). 20× magnification in areas without large TUNEL(+) regions is shown (B). Quantification of TUNEL(+) cells in MiaPaCa-2 tumors. Stars indicate p<0.001 (C). Quantification of TUNEL(+) cells in HT1080 tumors harvested at 28 days and processed similarly as MiaPaCa-2 tumors. Star indicates p<0.001 and # indicates p<0.05 (D).

Tissue sections from xenografts expressing different OPN constructs were stained for Ki67 to assess proliferation, CD31 for angiogenesis, CD11b for bone marrow-derived cell infiltration and TUNEL for apoptosis. Ki67, CD31, and CD11b staining were similar between all tumors (data not shown); however, there was a difference in TUNEL staining among the tumor groups. While there were numerous TUNEL(+) regions within the control and OPN-A-RAA tumor sections, these regions were absent in WT OPN-A expressing tumors (Fig. 2A–B). Quantitation of TUNEL(+) cells per high power field (HPF) confirmed these observations (Fig. 2C). Similar results were seen for the OPN-B isoforms, with fewer TUNEL(+) cells in WT OPN-B as compared to OPN-B-NoRGD tumors (Fig. 2D). These results indicate that both OPN isoforms inhibit apoptosis and that this inhibition is mediated through the RGD.

### Tumor-Derived Secreted OPN Can Suppress Apoptosis but Has No Effect on Cell Adhesion and Migration

**Figure 3:**
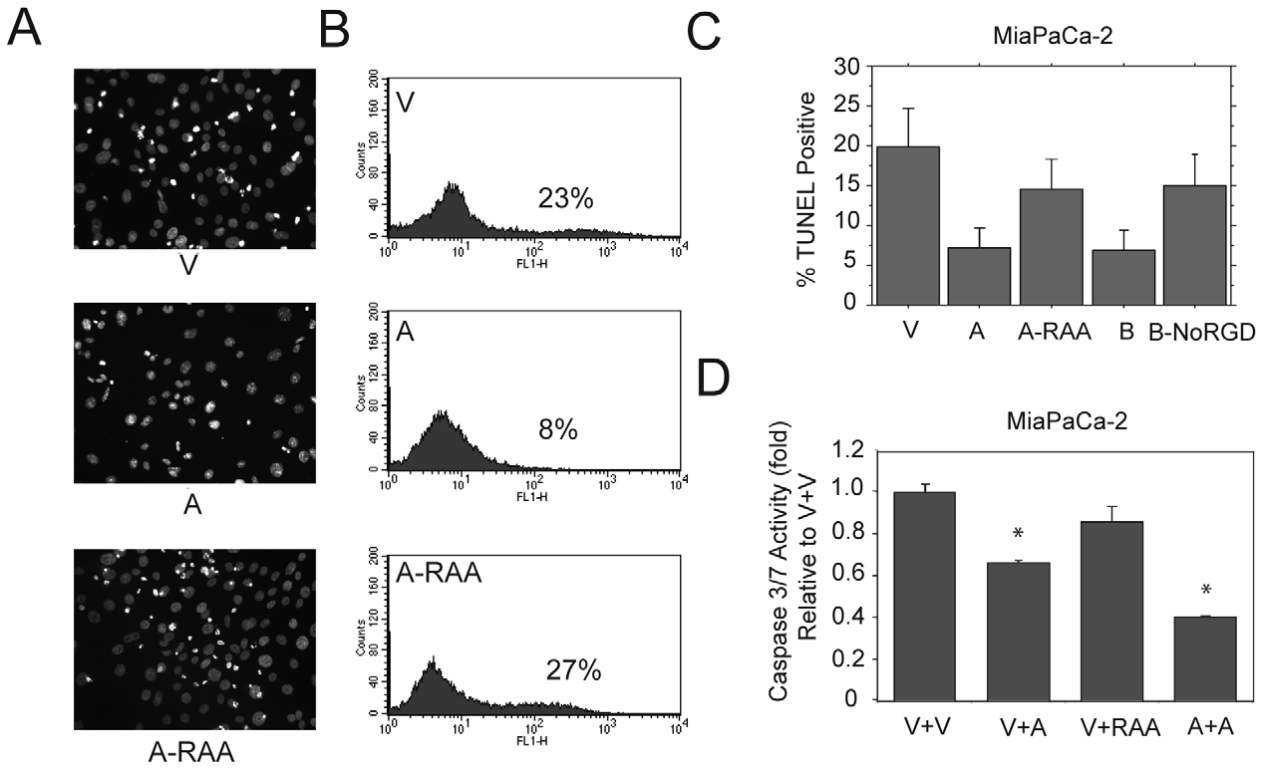
OPN prevention of apoptosis requires the RGD domain. MiaPaCa-2 cells transfected with the vector control (V), OPN-A (A) or OPN-A-RAA (A-RAA) were treated with hypoxia in serum free media for 48 hours and stained with DAPI. Note that more apoptotic nuclei are present in MiaPaCa-2 vector control and RAA compared to OPN-A overexpression (A). Histograms showing TUNEL(+) cells by flow cytometry after 48 hours of hypoxia in serum free media (B). Quantification of the percentage of TUNEL-positive cells in MiaPaCa-2 cells transfected with the indicated OPN constructs from flow cytometry, p<0.02 (C). Measurement of caspase 3/7 activity in MiaPaCa-2 vector control cells (V) treated with condition media harvested from vector (V+V), OPN-A (V+A) and OPN-A-RAA (V+RAA) secreting cells is shown. Caspase 3/7 activity for MiaPaCa-2 OPN-A overexpressing cells incubated in conditioned media from OPN-A secreting cells (A+A) is also indicated. Stars indicate p<0.0001 compared to V+V group (D).

Most solid tumors possess regions of reduced oxygen and nutrients. MiaPaCa-2 cells overexpressing different OPN constructs were treated with hypoxia in serum-free media for 48 hrs, which caused cell apoptosis in all groups. However, there were fewer apoptotic nuclei (Fig. 3A) and significantly less TUNEL(+) cells in WT OPN-A expressing cells as compared to vector control (Fig. 3B–C). Mutation of the RGD sequence to RAA prevented OPN-mediated protection of apoptosis from hypoxia and serum starvation (Fig. 3A–C). Similarly, OPN-B overexpression significantly reduced apoptosis under hypoxia and serum starvation in HT1080 cells, and deletion of the RGD sequence reversed this effect (Fig. S2). The conditioned media from WT OPN-A secreting cells, but not OPN-RAA cells, was able to reduce cell death from hypoxia and serum starvation when applied to vector control cells, assessed by Caspase 3/7 activity assay (Fig. 3D). These data indicate that OPN-A and B isoforms inhibit apoptosis induced by hypoxia and serum starvation through the RGD domain.

**Figure 4:**
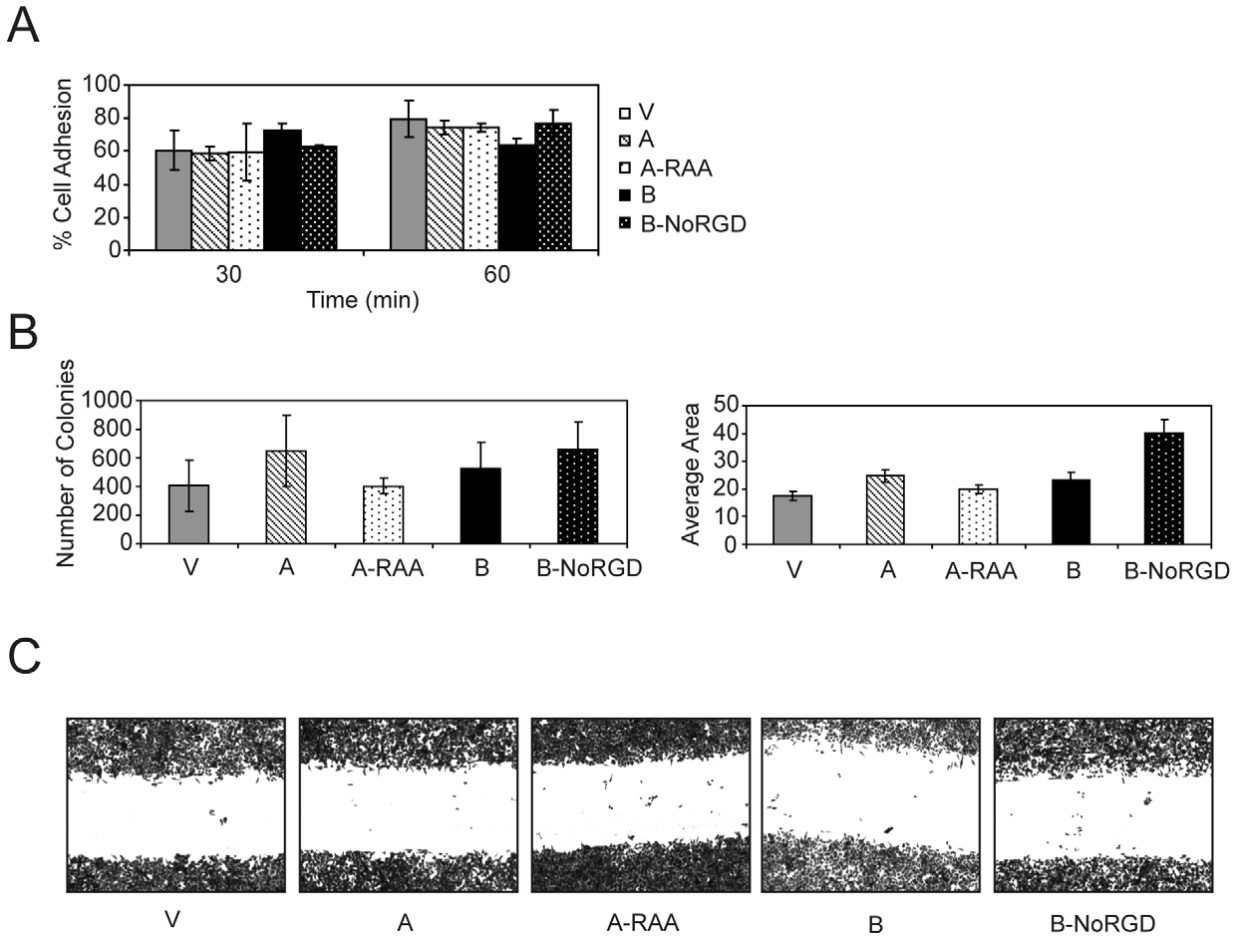
Overexpression of different OPN isoforms or their mutants does not affect cell-cell adhesion, soft agar colony formation or migration in serum free media. A cell-cell adhesion assay was performed with FaDu HNC cells (5×10^4^) expressing the indicated OPN constructs. Adherent cells were quantified by luciferase activity (A). Soft agar colony formation of MiaPaCa-2 cells overexpressing different OPN constructs was quantified by number of colonies (left graph) and the average colony area (right graph) (B). A Scratch assay was performed with MiaPaCa-2 cells overexpressing different OPN constructs exhibiting similar cell migration. Picture was taken at 40× total magnification (C).

To determine the effect of RGD domain on other cellular attributes that may enhance tumor growth or metastasis, we evaluated the effect of OPN overexpression, either WT or with the RGD mutated on cell-cell adhesion (serum free media) and anchorage independent growth (soft agar assay). Neither OPN overexpression or RGD mutation significantly influenced cell-cell adhesion or soft agar growth (assessed for both colony number and size) (Fig. 4A–B). Similarly, neither overexpression of OPN-A or B nor treatment of recombinant OPN isoforms increased cell migration or invasion in the tested cell lines in serum free media (data not shown). Scratch assays also showed that mutation of the RGD did not affect cell migration in serum free media (Fig. 4C). These studies confirm that the main function of the RGD domain is to protect cell from apoptosis.

### The RGD Domain of OPN Mediates Apoptosis Partially via NF-κB Activation

**Figure 5:**
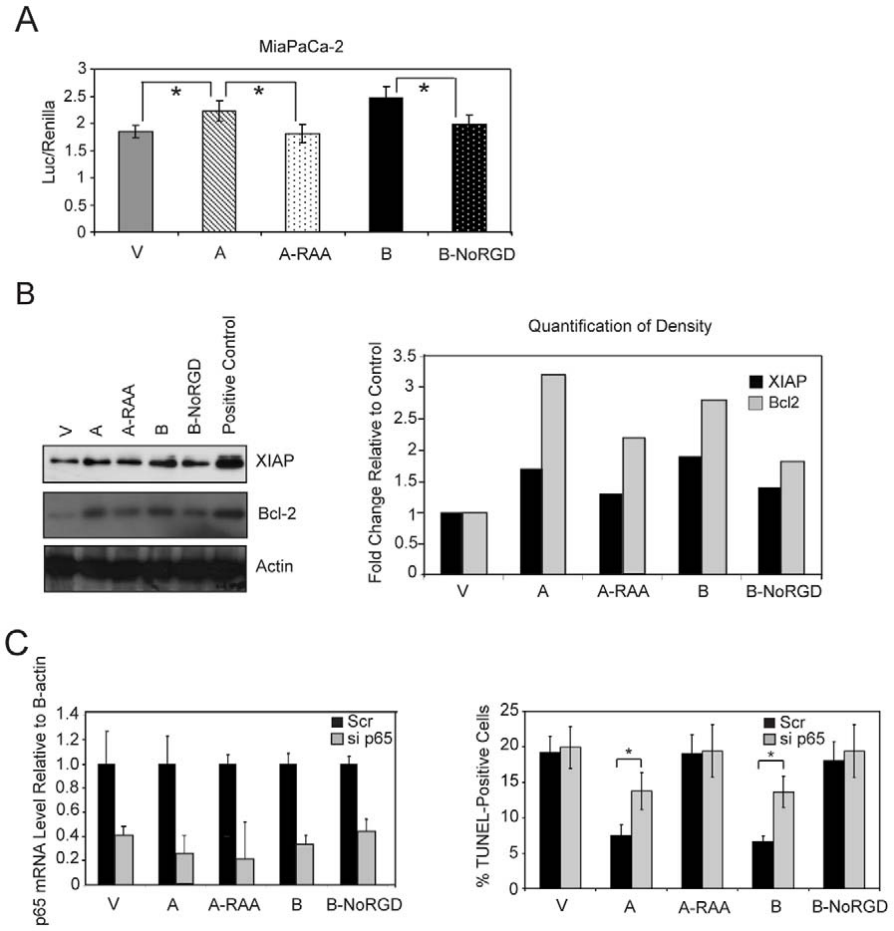
The RGD domain of OPN enhances NF-κB activation under stress conditions and partially protects cells from apoptosis. Quantification of NF-κB-dependent luciferase activity in MiaPaCa-2 cells overexpressing the indicated OPN constructs cultured under hypoxia and serum starvation. Stars indicate p<0.05 (A). XIAP and Bcl-2 immunoblots of MiaPaCa-2 cells overexpressing the indicated hOPN WT and hOPN mutants are shown. β-actin blot confirms equal protein loading (B, left blots). Quantitation of band intensities is shown (B, right graph). Inhibition of p65 expression reduces OPN-mediated cell survival (C). RT-PCR confirmation of similar p65 knockdown with p65-specific siRNA (grey bars) in MiaPaCa-2 cells stably transfected with the indicated constructs. Data was normalized to scramble control (black bars) (C, left graph). Flow cytometry quantification of TUNEL-positive staining MiaPaCa-2 cells, stably transfected with the indicated constructs and subjected to either p65 knockdown or scramble control, after 48 hours hypoxia in serum free media was performed. Stars indicate p<0.05. (C, right graph).

NF-κB activation protects cells from apoptosis and OPN can promote NF-κB activation. Hence, we evaluated NF-κB activity in MiaPaCa-2 cells expressing different OPN constructs. Overexpression of WT OPN isoforms resulted in significant increase of NF-κB-dependent luciferase activity and either mutation (OPN-A-RAA) or deletion (OPN-B-NoRGD) of the RGD domain eliminated this effect, although the absolute effect was modest (Fig. 5A). We also assessed protein expression of selective NF-κB endogenous targets. OPN-A or OPN-B overexpressing cells had higher Bcl-2 and XIAP protein levels than vector control, OPN-A-RAA and OPN-B-NoRGD cells (Fig. 5B). These results indicate that OPN activates NF-κB under stressed conditions, and the RGD domain of OPN mediates this effect.

MiaPaCa-2 cells overexpressing different OPN constructs were transiently transfected with a p65 siRNA oligo to inhibit NF-κB activation. Transfection with this oligo had minimal effect on apoptosis in control, OPN-A-RAA or OPN-B-NoRGD cells under stressed conditions (Fig. 5C). In contrast, inhibition of p65 increased the number of TUNEL(+) cells in those overexpressing either WT OPN-A or B compared to the scrambled control (13.8% vs. 7.5% and 13.6% vs. 6.6%, respectively, Fig. 5C). However, the level of apoptosis with NF-κB inhibition in WT OPN cells was still less than that observed in vector control or OPN-A-RAA cells, suggesting that NF-κB activation alone may only be partially responsible for OPN-mediated cell survival.

IκB Kinase β (IKK-β) activates NF-κB through phosphorylation and degradation of IκB-α. High IKK-β correlates with increased NF-κB activity and vice-versa. Although we cannot directly measure NF-κB activity in archival human cancer tissues, we can stain them for IKK-β as a surrogate marker for NF-κB activity. We stained a tissue microarray containing 89 HNSCCs for OPN and IKK-β. We found a significant correlation between OPN and IKK-β expression (p = 0.025, Fisher exact test). Concordant expression between the two markers was noted in 70 tumors and discordant expression in 19 tumors (data not shown). These results suggest that NF-κB activity and OPN expression are strongly related in human tumors.

### The RGD Segment of OPN Also Mediates Apoptosis Partially via FAK Activation

**Figure 6:**
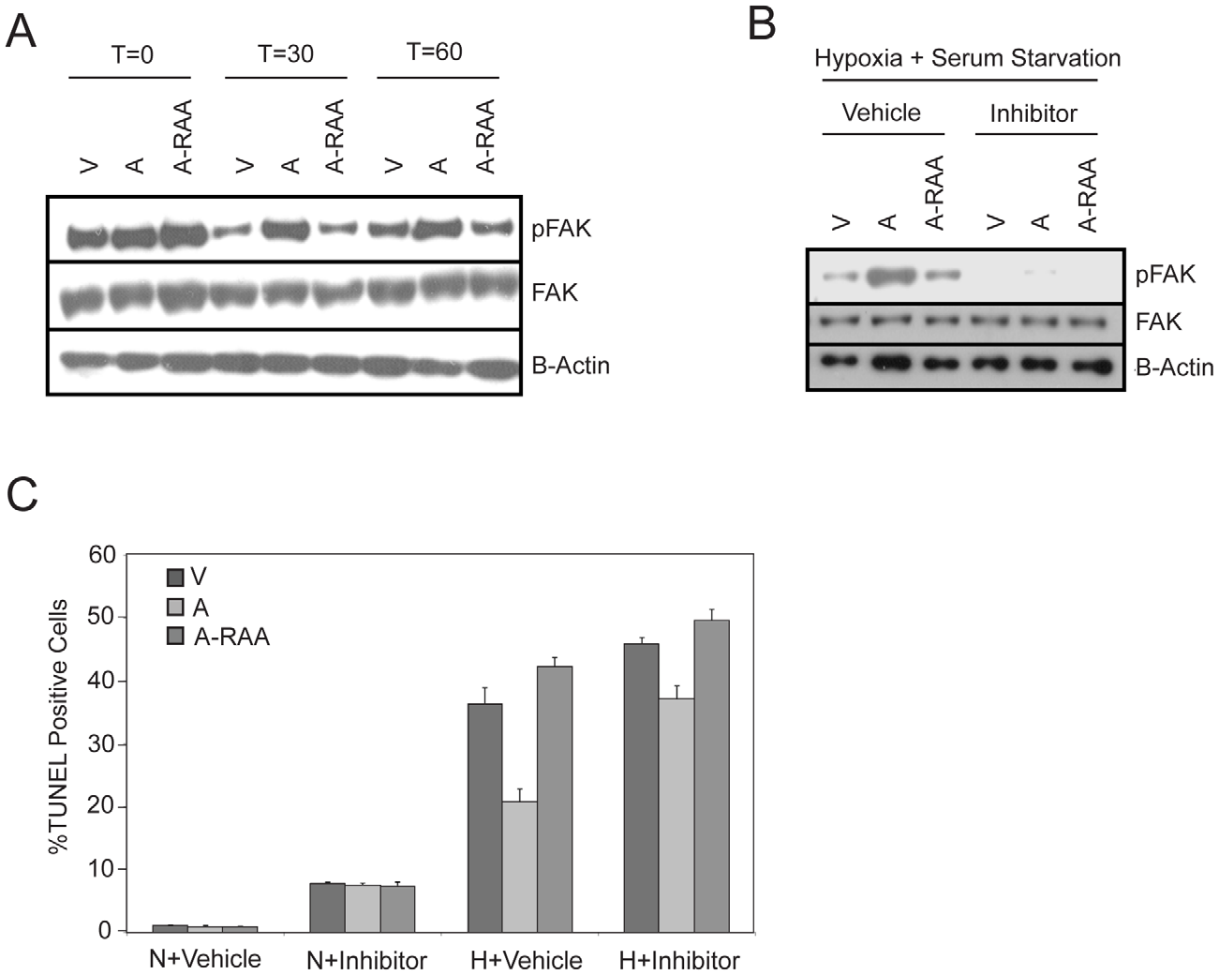
The RGD domain of OPN activates FAK and partially protects cells from apoptosis under stress conditions. OPN overexpression sustains FAK activation during stress through the RGD domain. Immunoblot analysis of phospho-FAK and total FAK in MiaPaCa-2 cells stably transfected with constructs for OPN-A (A), OPN-A-RAA (RAA), or vector control (V), treated with hypoxia in serum free media for the indicated times (minutes). β-actin blot shows equal loading (A). Addition of PF573228 (FAK inhibitor) completely inhibits FAK phosphorylation. Phospho-FAK and total FAK immunoblotting in MiaPaCa-2 cells (Vector, OPN-A, OPN-A-RAA) pre-treated with PF573228 (500 nM) or DMSO for 30 minutes followed by exposure to hypoxia for an additional 30 minutes. β-actin blot shows equal loading (B). Inhibition of FAK activation reduced OPN-mediated cell survival under stress. MiaPaCa-2 cells (Vector, OPN-A, OPN-A-RAA) were pre-treated with either vehicle or PF573228 (500 nM) for 30 minutes and then exposed to either normoxia or hypoxia in serum free media for 40 hours. TUNEL(+) cells were quantified by flow cytometry.

Since the RGD sequence binds to α_v_ integrins, which are known to signal through the FAK pathway, and OPN activates FAK in cell migration, we also evaluated the effect of RGD mutation on the activation of this pathway. MiaPaCa-2 cells expressing different OPN constructs were subjected to hypoxia and serum starvation. Prior to hypoxia and serum starvation, pFAK levels were elevated in all cells. After 30–60 min of hypoxia and serum starvation, FAK phosphorylation decreased in the vector control and OPN-A-RAA cells, whereas they remained high in WT OPN-A expressing cells (Fig. 6A). These results indicate that the RGD domain within OPN is important for sustaining FAK activation under stress.

To suppress FAK activation, MiaPaCa-2 cells expressing OPN isoforms were pre-treated with the FAK-specific inhibitor PF573228 (500 nM) or the vehicle for 30 min followed by exposure to hypoxia. As shown in Figure 6B, pretreatment with the FAK inhibitor completely blocked FAK phosphorylation at the tyrosine 397 residue in all cells.

As previously shown, overexpression of OPN-A or B did not enhance cell migration or invasion in evaluated cell lines. To determine if FAK phosphorylation is required for OPN-mediated cell survival under hypoxia and serum-starvation, MiaPaCa-2 cells expressing different OPN constructs were treated with PF573228 under these conditions. Addition of FAK inhibitor led to a small increase in TUNEL(+) cells under control conditions, suggesting a modest cytotoxic effect of the drug alone (Fig. 6C, N+Vehicle vs N+Inhibitor). However, under serum starved/hypoxia treatment, the addition of PF573228 strongly increased the percentage of TUNEL(+) cells for OPN-A expressing cells compared to vehicle (Fig. 6C). In contrast, the percentage of TUNEL(+) cells was only slightly elevated in control and OPN-A-RAA cells treated with PF573228. Taken together, these data suggest that OPN activates FAK through an RGD-dependent mechanism, which protects cells from apoptosis during hypoxia and serum starvation.

Since AKT has been shown to be activated by OPN and is a well-known survival factor, we also assessed AKT phosphorylation for cells transfected with different constructs under different conditions. There was no difference in the level of either total or phospho-AKT for cells expressing different constructs under hypoxia and serum starvation (Fig. S3).

## Discussion

The effect of modulating OPN expression has been studied in many cancer types. Overexpression of OPN increased growth [32], [33], whereas inhibition of OPN reduced growth in multiple tumor types [32], [34], [35]. These gain and loss of function studies indicate that OPN is involved in modulating tumor growth; however, the importance of the RGD domain within OPN in different OPN isoforms has not been rigorously characterized genetically. Here, we systematically define the effect of the RGD domain on tumor progression by either mutation or deletion in 2 different OPN isoforms and express them in different cell types. Overexpression of OPN isoforms containing the WT RGD, but not those with the RGD region mutated or removed, enhanced both local tumor growth and metastatic lung colony formation. In fact, the RGD domain was essential for tumor growth promotion in our xenograft models.

Interestingly, our mouse tumor data indicate that the RGD domain of OPN mediates tumor growth and metastasis mainly through decreased apoptosis. Similar findings were noted for *in vitro* assays. Overexpression of WT OPN but not mutant RGD protects cells from apoptosis triggered by hypoxia and serum starvation. However, such overexpression did not affect cell adhesion, migration or anchorage independent growth.

Although our results, which show that the major function of the RGD domain is to protect cancer cells from stress-induced apoptosis are different from prior studies using recombinant bacterially derived OPN [36], [37], [38], [39], they are consistent with studies which used human-derived soluble OPN or antibodies that block autocrine OPN function [40], [41], [42], [43]. OPN deficient mice have higher TUNEL staining and less papilloma development when treated with carcinogen compared to WT mice [44]. Reducing OPN expression by RNA interference leads to increased TUNEL staining and caspase-3 activity [45]. Mechanistically, OPN has been shown to counteract the pro-apoptotic effect of serum starvation in two-dimensional [40] and three-dimensional cultures [41], through the RGD domain. Interestingly, intracellular cleavage of OPN by caspase 3 and 8 can enhance apoptotic cell death under hypoxia and reoxygenation, and one of the cleavage sites is located immediately adjacent to the RGD [43]. Collectively, these reports suggest that OPN isoforms promote both tumor growth and metastasis through the same mechanism, which is to protect cells from apoptosis under the stresses encountered within the tumor microenvironment, notably through the RGD domain. Since the RGD domain of OPN interacts with multiple cellular receptors including the α_v_ family of integrins, strategies that block such an interaction may be beneficial in the treatment of solid tumors. For example, CNTO 95, a fully human anti-α_v_ integrin monoclonal antibody, has been shown to have antitumor activity by itself [46], [47] and can enhance the therapeutic efficacy of fractionated radiation therapy in xenografts via increasing apoptosis [48]. Several clinical studies are ongoing to test the toxicity and efficacy of such anti-integrin antibody either alone or in combination with conventional therapy in solid tumors.

OPN is well known in the literature as a survival factor that mediates cell apoptosis under certain pathological stresses. It has been shown to inhibit cell apoptosis in several systems including cardiac myocytes from ischemia/reperfusion injury [49], endothelial cells from growth factor deprivation [40], intestinal epithelial cells from inflammation [50] and immune cells from certain stresses [51], [52], [53]. However, the detailed pathways and mechanisms by which OPN mediates its anti-apoptotic effect can be different for various cell types and stressors. NF-κB activation is believed to be a major effector of OPN-mediated cell survival. OPN protects cells from denatured collagen II, by increasing p50 and p65 DNA binding [54]. Soluble OPN protects lymphocytes from apoptosis, by activating NF-κB [55]. Inhibition of OPN leads to increased cell death, increased TUNEL staining, decreased NF-κB binding and increased levels of the inhibitor IκB-α [34]. Furthermore, interaction between the RGD domain of OPN and integrin α_vβ3_ receptors leads to NF-κB activation and cell survival [56]. Our data confirm that overexpression of OPN isoforms containing the RGD domain lead to enhanced NF-κB activation, while isoforms lacking the RGD domain do not. Such activation does occur in actual human HNSCC as suggested by co-localization between OPN and IKK-β, a target of NF-kB pathway [57], [58]. Inhibition of NF-κB activity partially suppresses OPN-mediated protection from apoptosis in hypoxic/serum-starved cells. Our data therefore indicates that NF-κB activation is at least partially responsible for OPN mediated survival via the RGD domain.

FAK activation promotes cell proliferation, survival and migration [59]. FAK is tyrosine phosphorylated in response to ligation of β_1_ or β_3_ integrins [60]. Since the RGD domain of OPN binds to α_vβ3_ and α_vβ1_ integrins, FAK activation was measured in cell lines overexpressing OPN isoforms. WT OPN isoforms, but not those lacking the RGD domain, retained FAK phosphorylation in hypoxic/serum-starved cells. These results are similar to those previously published [61], [62]. Moreoever, inhibition of FAK phosphorylation with a specific inhibitor resulted in a marked enhancement of apoptosis seen only in WT OPN expressing cells but not those with mutated OPN-A isoform. Our data indicates that FAK is another survival pathway that is regulated by the RGD domain of OPN. However, not all survival pathways are engaged by the RGD domain of OPN as AKT is one such pathway (Figure S3) [63], indicating that RGD activation of survival pathways under hypoxia and serum starvation is a specific process.

Little is known about the function of different OPN isoforms in human tumors. The first study which systematically evaluated the expression and function of different OPN splice forms in breast cancer indicated that OPN-C was selectively expressed in invasive breast cancer and more likely to support anchorage independent tumor growth than OPN-A in a breast cancer cell line. However, subsequent studies have found that OPN-A and B are more likely to be expressed in hepatocellular carcinoma and lung cancer than OPN-C [64], [65]. Overexpression of OPN-A and to some extent OPN-B in NSCLC cell lines resulted in increased VEGF secretion and bovine capillary tube length formation, whereas the reverse was noted for OPN-C overexpression [64]. Similarly, OPN-A and B induced Hep3B cell migration, while OPN-C had no significant effects [65]. These data are consistent with our findings, indicating that OPN-A and B are the predominant isoforms found in solid tumors and that both can enhance tumor growth and metastasis at an equivalent level.

In summary, expression of both OPN-A and OPN-B isoforms are commonly observed in NSCLC and HNSCC. Forced overexpression of either isoform promotes tumor growth and metastasis, and the RGD domain within OPN is required for the effect to occur. OPN overexpressing cells have reduced apoptotic cell death both *in vivo* and *in vitro*. OPN, acting through the RGD domain, protects the cells by activating NF-κB and FAK but not AKT. These data indicate that the predominant mechanism, by which OPN promotes tumor growth and metastasis through the RGD domain, is enhancement of survival in the tumor microenvironment. Therefore inhibitors directed against OPN should target multiple isoforms and should inhibit cell survival mechanisms that involve the RGD domain, FAK phosphorylation and NF-κB activation.

## Supporting Information

Figure S1 Expression of OPN-A and OPN-B isoforms in human cancer cell lines and primary human tumors. Poly-A RNA was extracted from human STS including giant cell tumor (GCT), pigmented villonodular synovial tumor (PVNS) and leiomyosarcoma (LMS) samples and amplified by RT-PCR using the universal OPN primer set (left) (A). HT1080 RNA was used as a positive control for OPN-A and OPN-B expression (A, right lane). RNAs extracted from the indicated human cancer cell lines were subjected to RT-PCR and resulting cDNAs were amplified using the universal OPN primer set (right panel) (A). pCDNA -OPN-A and B plasmids were used as positive controls for the amplification of the indicated OPN isoforms. NS indicates non-specific band (A, right panel). The fold increase of NSCLC OPN-A (stripe) and OPN-B (black) mRNA expression relative to 18S in human NSCLC specimens (LCC) and Normal Lung Tissue (NOR)( B) or in human HNSCC normalized to HN99 (one HNSCC sample) (C), based on qRT-PCR assay is shown. Tumors were arranged by overall total OPN mRNA expression based on gene expression data with increasing expression from left to right (B and C).
(0.42 MB TIF)

Figure S2 Percentage of TUNEL(+) cells in HT1080 cells transfected with different OPN constructs under hypoxia in serum free media.
(0.10 MB TIF)

Figure S3 Immunoblot showing total AKT and pAKT expression for MiaPaCa-2 cells expressing different OPN constructs under normoxia or 1 hour of hypoxia in serum free media. β-actin bands confirmed equal loading.
(0.17 MB TIF)
