# Supplementary figures and images for: The RGD Domain of Human Osteopontin Promotes Tumor Growth and Metastasis through Activation of Survival Pathways
Source: PLoS One. 2010 Mar 10;5(3):e9633. doi: 10.1371/journal.pone.0009633 (PMC2835762; doi:10.1371/journal.pone.0009633)

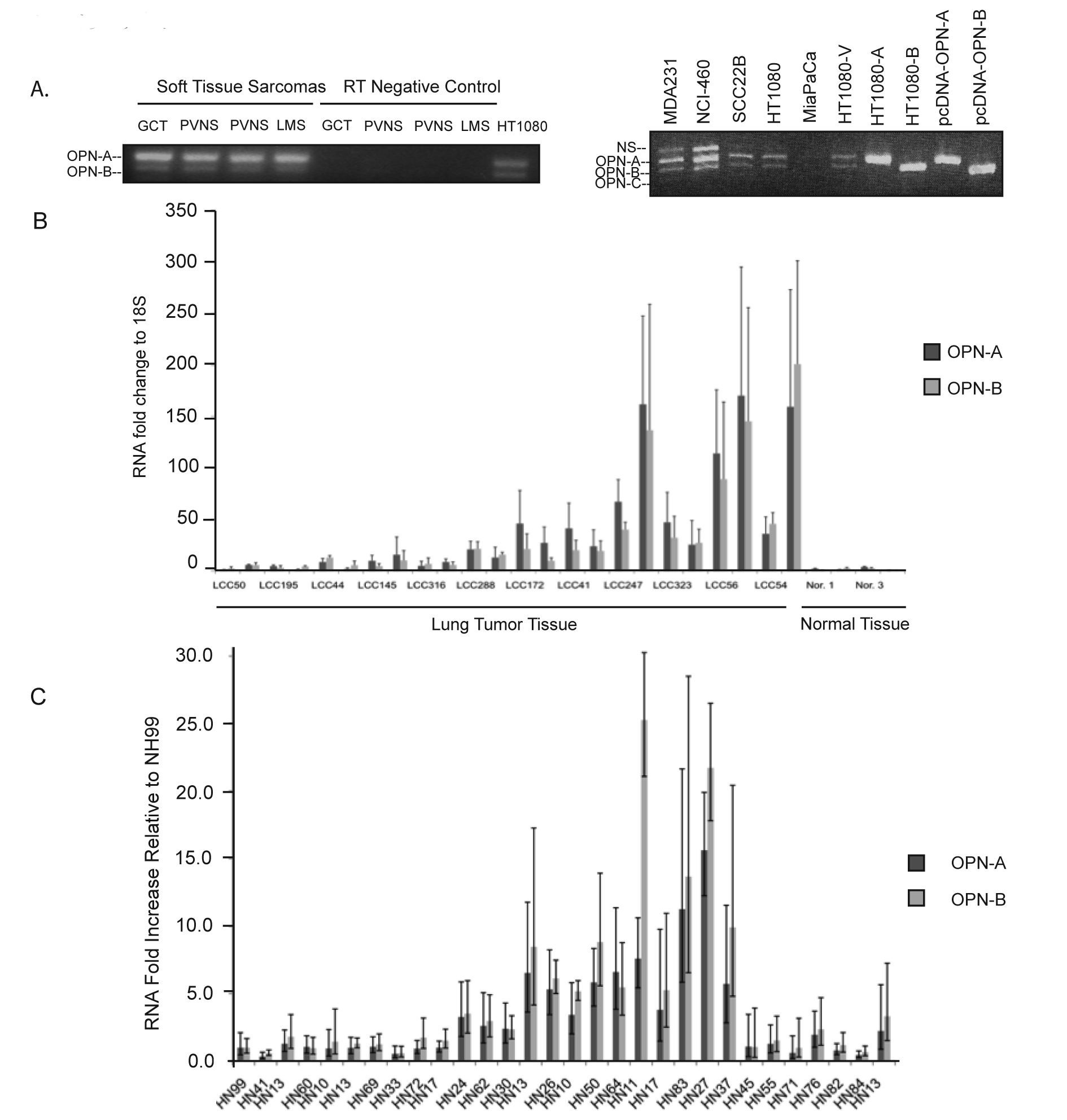

Supplement: Figure S1 — Expression of OPN-A and OPN-B isoforms in human cancer cell lines and primary human tumors. Poly-A RNA was extracted from human STS including giant cell tumor (GCT), pigmented villonodular synovial tumor (PVNS) and leiomyosarcoma (LMS) samples and amplified by RT-PCR using the universal OPN primer set (left) (A). HT1080 RNA was used as a positive control for OPN-A and OPN-B expression (A, right lane). RNAs extracted from the indicated human cancer cell lines were subjected to RT-PCR and resulting cDNAs were amplified using the universal OPN primer set (right panel) (A). pCDNA -OPN-A and B plasmids were used as positive controls for the amplification of the indicated OPN isoforms. NS indicates non-specific band (A, right panel). The fold increase of NSCLC OPN-A (stripe) and OPN-B (black) mRNA expression relative to 18S in human NSCLC specimens (LCC) and Normal Lung Tissue (NOR)( B) or in human HNSCC normalized to HN99 (one HNSCC sample) (C), based on qRT-PCR assay is shown. Tumors were arranged by overall total OPN mRNA expression based on gene expression data with increasing expression from left to right (B and C). (0.42 MB TIF) [file pone.0009633.s001.tif]

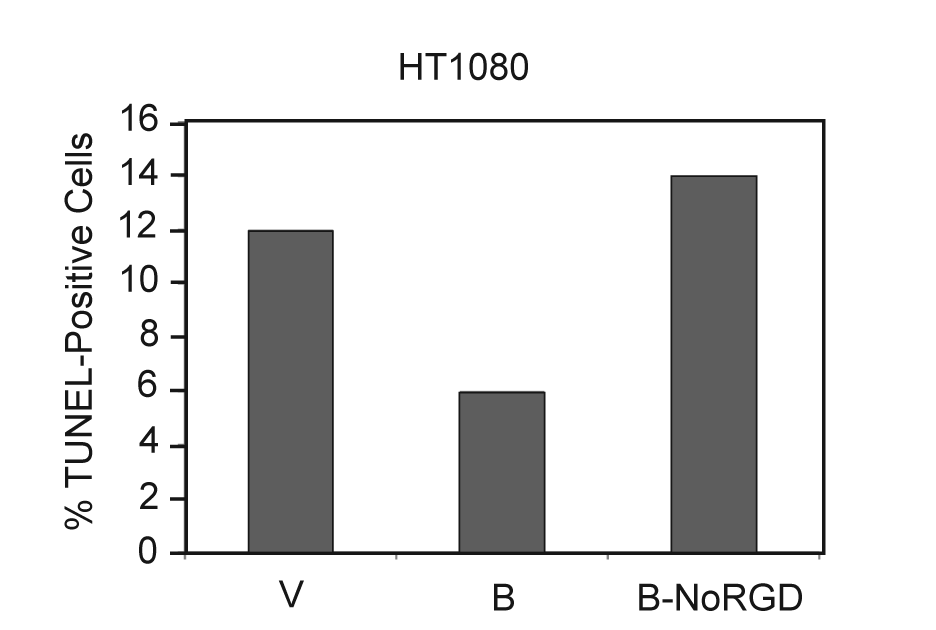

Supplement: Figure S2 — Percentage of TUNEL(+) cells in HT1080 cells transfected with different OPN constructs under hypoxia in serum free media. (0.10 MB TIF) [file pone.0009633.s002.tif]

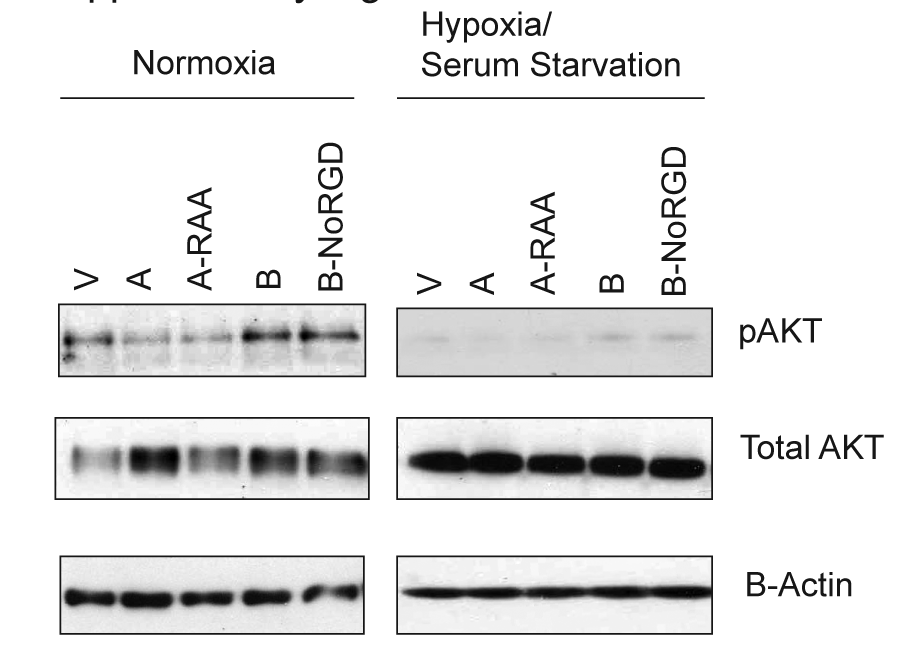

Supplement: Figure S3 — Immunoblot showing total AKT and pAKT expression for MiaPaCa-2 cells expressing different OPN constructs under normoxia or 1 hour of hypoxia in serum free media. β-actin bands confirmed equal loading. (0.17 MB TIF) [file pone.0009633.s003.tif]
